# Guide to clinical practice guidelines: the current state of play

**DOI:** 10.1093/intqhc/mzv115

**Published:** 2016-01-21

**Authors:** Tamara Kredo, Susanne Bernhardsson, Shingai Machingaidze, Taryn Young, Quinette Louw, Eleanor Ochodo, Karen Grimmer

**Affiliations:** 1 South African Cochrane Centre (SACC), South African Medical Research Council, Francie van Zijl Drive, Parow Valley, Cape Town 7505, South Africa; 2 Närhälsan Rehabilitation, Region VästraGötaland, Hönö, Sweden; 3 Department of Medical and Health Sciences, Division of Physiotherapy, Linköping University, Linköping, Sweden; 4 Centre for Evidence-Based Health Care (CEBHC), Faculty of Medicine and Health Sciences, Stellenbosch University, Francie van Zijl Drive, Tygerberg, Cape Town 7505, South Africa; 5 Department of Physiotherapy, Faculty of Medicine and Health Sciences, Stellenbosch University, Francie van Zijl Drive, Tygerberg, Cape Town 7505, South Africa; 6 International Centre for Allied Health Evidence (iCAHE), University of South Australia, City East Campus, P4-18 North Terrace, Adelaide 5000, Australia

**Keywords:** clinical practice guidelines, guideline development, implementation, adaptation

## Abstract

**Introduction:**

Extensive research has been undertaken over the last 30 years on the methods underpinning clinical practice guidelines (CPGs), including their development, updating, reporting, tailoring for specific purposes, implementation and evaluation. This has resulted in an increasing number of terms, tools and acronyms. Over time, CPGs have shifted from opinion-based to evidence-informed, including increasingly sophisticated methodologies and implementation strategies, and thus keeping abreast of evolution in this field of research can be challenging.

**Methods:**

This article collates findings from an extensive document search, to provide a guide describing standards, methods and systems reported in the current CPG methodology and implementation literature. This guide is targeted at those working in health care quality and safety and responsible for either commissioning, researching or delivering health care. It is presented in a way that can be updated as the field expands.

**Conclusion:**

CPG development and implementation have attracted the most international interest and activity, whilst CPG updating, adopting (with or without contextualization), adapting and impact evaluation are less well addressed.

## Introduction

High-quality, evidence-informed clinical practice guidelines (CPGs) offer a way of bridging the gap between policy, best practice, local contexts and patient choice. Clinical guidelines have been upheld as an essential part of quality medical practice for several decades. An early definition of CPGs by the Institute of Medicine (IOM) [1] described it as ‘systematically developed statements to assist practitioner and patient decisions about appropriate health care for specific clinical circumstances.’ This definition was updated in 2011 to more strongly emphasize rigorous methodology in the guideline development processes: ‘Clinical guidelines are statements that include recommendations intended to optimize patient care that are informed by a systematic review of evidence and an assessment of the benefits and harms of alternative care options’ [2]. In this rapidly evolving field of research, a more recent definition suggested a modern twist to the guideline description: ‘Guidelines are a convenient way of packaging evidence and presenting recommendations to healthcare decision makers’ [3].

Guidelines have a range of purposes, intended to improve effectiveness and quality of care, to decrease variations in clinical practice and to decrease costly and preventable mistakes and adverse events. They generally include statements of expected practice; provide benchmarks or standards against which individuals can audit; compare and potentially improve their practices; or guidance regarding undertaking particular tasks [4, 5]. Quality improvement initiatives are linked with CPGs, as evidence-informed recommendations form the basis for identifying core outcomes and measurable standards of care [6]. Internationally, over the past decade in particular, an industry seems to have developed around CPG development, reporting, adoption, contextualization or adaptation, evaluation and implementation. The growing volume of evidence and the acronyms used in this field can be overwhelming, even for those involved. This article is targeted at individuals and organizations working in health care quality and safety; and responsible for either commissioning, researching or delivering health care. We aim to provide a guide describing common standards, methods and systems used in current international CPG activities and the various activities to produce and communicate them.

## Terminology

Guidelines, CPGs, protocols and care pathways are commonly used terms, but without common agreement about their definitions [7]. Definitions that we have found useful are that *guidelines* relate to broader systems, such as those found in primary care (e.g. water or air quality, food security, incident reporting and investigation, etc.) and are generally developed and used by policy-makers, service organizations, funders or regulatory authorities. *CPGs* relate to clinical matters, generally dealing with clinical conditions or symptoms, and are typically intended for use by health care providers and clinic managers [4]. They can include best-practice statements for any one or combination of concerns regarding screening, diagnosis, management or monitoring. The term ‘protocol’ is commonly used to prescribe behaviours at diplomatic and societal events. In health, it has the meaning of rules or instructions about how to do a particular process explicitly, and without error. *Care pathways* generally relate to a series of evidence-informed steps, which can involve a multidisciplinary team at various care levels (i.e. primary, secondary), which should underpin the journey of care of patients with a particular diagnosis [8, 9]. Whilst broadly similar to CPGs, clinical pathways differ by being more explicit about the sequence, timing and provision of interventions. They are usually based on CPGs and contextualized for use within specific environments or circumstances [9].

## Development

There are detailed processes available for developing a CPG. Notably, there are well-credentialed international and national guideline development groups, including the World Health Organization (WHO) [10], the Scottish Intercollegiate Guidelines Network (SIGN) [11], the National Institute for Health and Care Excellence (NICE) [12] and the Australian National Health and Medical Research Council (NHMRC) [13], each with their own approach to guideline construction and writing, usually described in a guideline development manual.

Globally, potentially many hundreds more health departments, insurers and other health care organizations, professional associations, hospitals, specialty colleges and individuals have attempted to produce recommendations to improve and/or standardize local clinical practices, all using their own interpretations of the best way to construct and write CPGs. The most common approach to CPG development seems to come from the efforts of small teams of dedicated volunteers, often working with minimal funding and variable understanding of CPG development methods, to produce recommendations for practice in local settings, based on a range of evidence sources. These include peer-reviewed literature, grey literature, other CPGs and expert opinion. Historically, CPGs were built mostly on expert opinion, which included variable (and often selective) reference to research evidence [14, 15]. Such CPGs are still found today, albeit in decreasing numbers, as transparently constructed evidence-informed approaches integrated with expert opinion and patient values have rapidly gained acceptance over the past two decades as the best approach to CPG development [14, 15]. To add to the complexity of the evolution of CPG development, developers around the world have used a range of different and purpose-built approaches to identify, appraise, synthesize and describe the evidence base underpinning best-practice statements. Thus, there is no standard approach to any aspect of CPG activity.

However, evidence of a maturing CPG development culture internationally is seen in recent attempts to standardize practices. In 2011, the Institute of Medicine (IOM) introduced eight standards for CPG development [16], which are similar to those promoted by the Guidelines International Network (G-I-N) [17] (Table 1).

**Table 1:** Comparing the elements of clinical practice guideline development between the Institute of Medicine (IOM) and the Guidelines International Network (G-I-N)

IOM [2]	Guidelines International Network (G-I-N) [17]
Standard 1: Establishing transparency	1: Composition of Guideline Development Group
Standard 2: Management of conflict of interest	2: Decision-making Process
Standard 3: Guideline development group composition	3: Conflicts of Interest
Standard 4: Clinical practice guideline – systematic review intersection	4: Scope of a Guideline
Standard 5: Establishing evidence foundations for and rating strength of recommendations	5: Methods
Standard 6: Articulation of recommendations	6: Evidence Reviews
Standard 7: External review	7: Guideline Recommendations
Standard 8: Updating	8: Rating of Evidence and Recommendations
	9: Peer Review and Stakeholder Consultations
	10: Guideline Expiration and Updating
	11: Financial Support and Sponsoring Organisation

In addition, a recent enterprise, conducted by McMaster University, systematically and comprehensively reviewed the methodological content of 35 international CPG development manuals, to identify key CPG development components. This work included the G-I-N and IOM criteria. The McMaster Group developed a checklist of 18 topics and 146 items [18]. This project, Guidelines 2.0, itemized all potentially relevant CPG steps, linked to primary resources and is able to be contextualized or adapted to local contexts. This provides a comprehensive resource; however, given the extensive list of items included, it may not be user-friendly. In another example of efforts to standardize methods, a step-by-step manual was developed to assist CPG developers in the area of head and neck cancer surgery [19].

Given these widely available best-practice approaches to CPG development that are now available to all, it seems sensible to reconsider the need for future *ad hoc* CPG development that does not comply with recommendations from at least one of these approaches [16]. Moreover, there is a wealth of freely accessible, good-quality CPGs from internationally respected development agencies [9–12] that can be adopted and then configured to meet local needs, using emerging CPG contextualization or adaptation methods (refer to ‘adopting, contextualising, adapting’ section) [10–13]. Thus there seems little merit in producing new CPGs, unless a true gap exists in available guidance. This gap should be verified by a comprehensive search of CPG repositories before any *de novo* activities take place. Where *de novo* CPGs are required, there are many comprehensive evidence-synthesis resources available (such as the Cochrane database of systematic reviews), which should make the CPG development processes less demanding. Given these efficiencies in sourcing the research evidence, the key issues for discussion by the development teams could then be oriented to the use and inclusion of local contextualized evidence regarding resource requirements, feasibility, cultural issues, patient preferences, values and approaches for shared decision-making.

## Determining the strength of the body of evidence

A critical methodological quality issue in CPG development is how best to describe the strength of the evidence underpinning recommendations. Numerous approaches to grading evidence have been developed. However, in the last few years, two main approaches have emerged to support systematic and comprehensive evidence synthesis: Grading of Recommendations Assessment, Development and Evaluation (GRADE) [20–23] and the Australian NHMRC approach, Formulating Recommendations Matrix (FORM) [24]. The GRADE approach has gained momentum internationally, with acceptance by, among other organizations, the WHO's Guideline Review Committee [10]. The GRADE and FORM approaches not only assist CPG developers to summarize the evidence body for a recommendation and consider its local relevance but also provide advice on how to proceed from evidence to recommendations in a standardized and transparent manner.

## Quality appraisal

Similar to evidence grading, a number of tools have been developed to support critical appraisal of CPG quality. Many of them have focused on structural issues such as the composition of the CPG team, the review dates, the layout and the CPG purpose and end use, whilst others focus on rigour of methodological development and applicability [25–27]. The AGREE II instrument (Appraisal of Guideline ResEarch and Evaluation) [28, 29] emerged internationally five years ago. It comprises six domains with a total of 23 items, each scored 1–7 (Strongly Disagree through to Strongly Agree). More than one scorer is required to determine a valid score, and a scoring rubric is required to combine scores into one composite score for each domain. A new, simplified tool, the iCAHE CPG quality checklist, was recently developed as an alternative to the AGREE approach [30]. The iCAHE instrument items were based on perspectives of CPG quality of busy clinicians, educators and policy-makers. It has similar domains to AGREE II, but only 14 questions, each with a binary response (Yes/No), requiring one scorer, and the overall score is the sum of the ‘Yes’ responses. Both instruments include questions regarding the CPG process, that is, the identification and reporting of the body of evidence underpinning the CPG. The two instruments show moderate to strong correlation in pilot testing (*r* = 0.89) with the iCAHE tool requiring significantly less time to administer.

## Updating

Considering the substantial international effort invested in CPG development, there has been much less research into the process of CPG updating. Whilst the importance of updating is noted in most CPG development manuals, specific processes for doing so are poorly described [31]. Examples of guidance on updating from the G-I-N and IOM development standards are provided in Table 2.

**Table 2:** Examples of guidance for updating from the Institute of Medicine (IOM) and the Guidelines International Network (G-I-N)

IOM STANDARD 8: Updating [2]	Guidelines International Network (G-I-N) [17]
The CPG publication date, date of pertinent systematic evidence review, and proposed date for future CPG review should be documented in the CPG. Literature should be monitored regularly following CPG publication to identify the emergence of new, potentially relevant evidence and to evaluate the continued validity of the CPG. CPGs should be updated when new evidence suggests the need for modification of clinically important recommendations. For example, a CPG should be updated if new evidence shows that a recommended intervention causes previously unknown substantial harm, that a new intervention is significantly superior to a previously recommended intervention from an efficacy or harms perspective, or that a recommendation can be applied to new populations.	A guideline should include an expiration date and/or describe the process that the guideline groups will use to update recommendations. Guidelines become outdated at different rates depending on the availability of new evidence. Therefore, it is important to identify the expiration date of a guideline, as well as an update process, if planned. Developers should prospectively determine whether and when they will update a guideline or when it should be considered inactive if an update is not performed.

A recently published systematic review aimed to identify best practices for updating CPGs [31]. The review authors systematically identified and appraised 35 CPG development handbooks which included information on CPG updating. They concluded that the available guidance on updating processes was lacking in detail, used variable terminology, and that more rigorous and explicit guidance would increase the trustworthiness of updated CPGs. This review did not include the systematic approach published in 2003 by Johnston *et al.* from the Cancer Care Ontario Practice Guidelines Initiative, which reports four criteria for use after an updated literature review has been performed. These criteria provide clear guidance regarding how recent literature might alter the earlier strength of the body of evidence (p. 648) (Table 3) [32]. These criteria have been used for the last three updates of the Acute pain management CPG by the Australian and New Zealand College of Anaesthetists and Faculty of Pain Medicine [33].

**Table 3:** Clinical Practice Guideline Update elements [32]

1	The new evidence is consistent with the data used to inform the original practice guideline report. The recommendations in the original report remain unchanged.
2	The new evidence is consistent with the data used to inform the original practice guideline report. The strength of the recommendations in the original report has been modified to reflect this additional evidence.
3	The new evidence is inconsistent with the data used to inform the original practice guideline report. However, the strength of the new evidence does not alter the conclusions of the original document. Recommendations in the original report remain unchanged.
4	The new evidence is inconsistent with the data used to inform the original practice guideline report. The strength of the new evidence will alter the conclusions of the original document. Recommendations in the original report will change. This change is a priority for the working party members. Modifications to the guideline are in progress.

Technologies for ‘dynamic updating’ of CPGs are also emerging [34]. The GRADE group is currently piloting an international collaborative initiative in CPG writing with corresponding implementation plans, aimed at ready implementation of recommendations – DECIDE: Developing and Evaluating Communication strategies to support Informed Decisions and practice based on Evidence [3]. This Consortium has supported the development of two interactive CPG development tools, the GDT (http://gdt.guidelinedevelopment.org/) [35] and ‘Making GRADE the Irresistible Choice’ MAGICapp (http://www.magicapp.org/) [36]. These multi-layer development and dissemination software tools could put up-to-date CPGs literally ‘in the pockets’ of clinicians via smartphones and tablets. These tools also allow for dynamic updating of evidence sources, and integration of evidence with electronic medical record tools [34].

## Presentation and communication

Concurrent with the evolution of standardized CPG development principles, there has been increasing interest in the manner in which recommendations are *written* and *presented* to best support uptake. This interest has stemmed from concerns with the need to address structural barriers to CPG uptake, in the way recommendations are worded and presented, as well as external barriers to implementation such as access and relevance [37]. To address this, a specific tool was developed for CPG developers and implementers (GuideLine Implementability Appraisal (GLIA)) that provided 10 dimensions of 31 items, including decidability and executability, global, presentation and formatting, measurable outcomes, apparent validity, flexibility and effect on process of care [38]. The DECIDE consortium is exploring methods to ensure effective communication of evidence-based recommendations targeted at key stakeholders: health care professionals, policy-makers and managers, as well as patients and the general public. Their multi-layer development and dissemination software tools allow one-click adaptation of display of content depending on the audience [3].

## Implementation

Another recently launched tool, GUIDE-M, is intended to enhance quality, implementability and acceptability of CPGs, the ‘Guideline Implementability for Decision Excellence Model’ (www.guide-m.ca) [39]. This tool was developed to reflect an evidence-informed, international and multidisciplinary perspective to putting CPGs into practice.

There is surprisingly little decisive guidance on how CPGs can be successfully *implemented*, and the knowledge gap regarding the effectiveness of CPGs on patient health outcomes is substantial. More is known about the effectiveness of various implementation strategies on process outcomes (how the system works) rather than clinical outcomes, although this impact is often modest [37, 40]. An overview by Grimshaw (2012) showed effects of evidence implementation strategies (not specific to CPGs) such as educational measures, audit and feedback, opinion leaders and tailored interventions, which resulted in 4.3–12% in median absolute improvements in care [41]. CPG implementation often requires behaviour change by health care professionals, patients and other stakeholders within the health care system, because they may need to change or discard ‘usual’ practices in light of current best-evidence recommendations.

CPG recommendations often include the introduction of new technologies or interventions or discontinuation of ineffective, costly or harmful interventions. To do this requires significant and often swift changes in clinician behaviour. For behaviour change to be successful, consideration of the context in which the CPG is to be used is paramount [42–44]. Several implementation theories account for context explicitly, e.g. the Promoting Action on Research Implementation in Health Services framework [45], the Consolidated Framework for Implementation Research [46] and the Theoretical Domains Framework (TDF) [47, 48]. The TDF is a validated framework that includes 14 domains of theoretical constructs and has been tested for developing complex interventions to implement changes in health care settings [49].

Theoretical frameworks of implementation can facilitate planning and executing implementation of CPG recommendations, as well as support evaluation of CPG impact [50–53]. However, few published CPG implementation interventions use specific theories. A recent systematic review reported that only one-fifth of the 235 CPG implementation studies reviewed used a specific theory [54]. Moreover, critics of implementation theories have highlighted the poor evidence supporting them and suggested that a common-sense approach may do just as well [55, 56]. However, there seems to be emerging evidence that behaviour-change processes applied in CPG implementation, that are informed by theory are more effective than those that are not and that theory should be used to establish causal relationships between theoretical constructs and effects of aspects of implementation [56, 57]. Further research is required to understand the practical aspects of how CPG recommendations can be effectively and efficiently implemented in ways that produce improvements in processes and clinical outcomes.

## Configuring CPGS to different settings: adopting, contextualizing or adapting

Since the early 2000s, there has been increasing international recognition of the potential for efficiency and value of taking CPGs developed in one country and applying them to other countries. This is intended to avoid duplication of effort in *de nov*o guideline development, when useful CPGs may exist elsewhere [26, 58]. There is no consensus on the appropriate terminology to use for transferring CPGs from one health system or health setting to another, or for subsequent configuration of CPGs for local contexts and needs. The ADAPTE Collaboration, a strategic collaboration between two international CPG research groups (ADAPTE and Practice Guideline Evaluation and Adaptation Cycle) proposes an ‘adaptation’ approach in their resource manual (distributed via G-I-N (ADAPTE Collaboration 2009)) [59]. Their work describes the direct transfer of CPGs across similar income and health systems settings.

Another approach, that of adopting and then contextualizing, underpinned an innovative Filipino CPG implementation project [60]. The ADAPTE process lacked detail on the specifics of ‘how to’ transfer recommendations from CPGs developed in high-income to low-income country settings, where health care policy and contexts, funding, workforce, resources and training are significantly different. The CPG working group from the Philippines Academy of Rehabilitation Medicine differentiated between the notions of ‘adaptation’ and ‘contextualization’ and proposed an innovative adoption and contextualization approach, by mapping recommendations from multiple CPGs into a typical Filipino patient pathway, and then developing local ‘context points’ to support local uptake [61]. This work has since been recognized as best practice for lower- and middle-income countries by the *International Society of Physical and Rehabilitation Medicine (ISPRM)* and provides a practical, cost-effective and efficient alternative approach to developing local context *de novo* CPGs.

## Shared decision-making

Shared decision-making occurs when patients and their health care providers make joint decisions about health care interventions based on best research evidence, and layered by patient preferences, values, clinical judgement and local contexts [62, 63]. When done well, shared decision-making and mutual agreement on the way forward for the management of a patient's condition could be considered the desired end-point of CPG implementation [62, 64]. Where high-quality evidence is lacking, shared decisions will rely more heavily on clinician perspectives and patient preferences [65]. Barriers to effective shared decision-making include lack of time, skills, knowledge, mutual respect and effective communication processes [63, 66]. A Cochrane review evaluating shared decision-making interventions reported low-quality evidence for the effectiveness of any intervention targeting health care professionals, patients or both. However, the authors conclude that despite the low-quality evidence, any intervention targeting both parties is consistently better than targeting either one or no intervention [63].

Decision aids are tools designed specifically to help with decision-making, with particular application in the context of low-quality or uncertain evidence [66]. These tools have been reported to increase absolute knowledge of patients amongst other benefits; however, effects on clinical outcomes are to date uncertain [67]. Rapid developments in evidence mean that decision aids may be out-of-date, and the process for updating may be onerous and, in many cases, not done [66]. There is a move to use new technology to support this process. Point-of-care decision aids include short one-page summaries as in ‘Option Grids’ (www.optiongrid.co.uk) [68]. Technology in development includes the previously mentioned MAGICapp group, where the layered approach extends to patient end-user tools for use in consultation, linked with the SHARE-IT project evaluating the value of the decision aid in clinical care (http://magicproject.org/share-it/) [69].

## Conclusion

This paper explores the standards, methods and systems in use by those involved with CPGs and provides a synthesis of the current state of play of international guideline activity. It also highlights the immense efforts being made by researchers, clinicians and policy-makers who are committed to optimizing ways in which evidence is packaged to improve care.

The tools described in this paper are not all uniformly accessible or user-friendly. They have variable evidence of psychometric properties and utility, and many require additional research to ensure that they can be applied appropriately in different CPG contexts.

CPG activities are evolving processes. We anticipate that the next decade will see significant further research into tools to underpin best practices in CPG activities. Given the increasing number of high-quality CPGs that are freely available internationally for a range of health conditions, we propose that the growth areas in CPG methods in the next decade will be in updating, adopting, contextualizing and/or adapting, and implementing. Moreover, the next generation of CPG activities should build on knowledge of current activities in development, advance processes of end-user engagement, and evaluate CPG impact on health outcomes.

## Authors’ contribution

K.G. lead the design and execution of the paper. Q.A.L., T.Y., T.K., S.M., S.B. and E.O. contributed to the conception or execution of the paper. All authors approved the final version

## Funding

This project was supported by the South African Medical Research Council Flagship Grants, 2014–2017 for the project South African Guidelines Excellence (SAGE), Cochrane South Africa, South African Medical Research Council.
